# Efficacy and pitfalls of digital technologies in healthcare services: A systematic review of two decades

**DOI:** 10.3389/fpubh.2022.869793

**Published:** 2022-09-16

**Authors:** Nadeem Akhtar, Nohman Khan, Shazia Qayyum, Muhammad Imran Qureshi, Snail S. Hishan

**Affiliations:** ^1^School of Urban Culture, South China Normal University, Foshan, China; ^2^UniKL Business School, Universiti Kuala Lumpur, Kuala Lumpur, Malaysia; ^3^Institute of Applied Psychology, University of the Punjab, Lahore, Pakistan; ^4^Teesside University International Business School, Middlesbrough, United Kingdom; ^5^Azman Hashim International Business School, Universiti Teknologi, Kuala Lumpur, Malaysia; ^6^Independent Researcher, THRIVE Project, Brisbane, QLD, Australia

**Keywords:** healthcare, digital technologies, artificial intelligence, IoT, blockchain, SLR-M

## Abstract

The use of technology in the healthcare sector and its medical practices, from patient record maintenance to diagnostics, has significantly improved the health care emergency management system. At that backdrop, it is crucial to explore the role and challenges of these technologies in the healthcare sector. Therefore, this study provides a systematic review of the literature on technological developments in the healthcare sector and deduces its pros and cons. We curate the published studies from the Web of Science and Scopus databases by using PRISMA 2015 guidelines. After mining the data, we selected only 55 studies for the systematic literature review and bibliometric analysis. The study explores four significant classifications of technological development in healthcare: (a) digital technologies, (b) artificial intelligence, (c) blockchain, and (d) the Internet of Things. The novel contribution of current study indicate that digital technologies have significantly influenced the healthcare services such as the beginning of electronic health record, a new era of digital healthcare, while robotic surgeries and machine learning algorithms may replace practitioners as future technologies. However, a considerable number of studies have criticized these technologies in the health sector based on trust, security, privacy, and accuracy. The study suggests that future studies, on technological development in healthcare services, may take into account these issues for sustainable development of the healthcare sector.

## Introduction

Technology has become an integral part of the healthcare sector and entirely transfigures medical practices. Cutting-edge digital technologies have improved the effectiveness of surgeries and helped maintain the quality of patient's life. Even individuals with severe medical complexities can maintain their health with the help of these technologies (1). The involvement of Artificial Intelligence (AI), machine learning, the Internet of Things (IoT), and blockchains revolutionized the healthcare sector, and the application of these technologies is beyond expected boundaries. The most promising advanced usage of these technologies is robotic surgery which has proved to be more efficient than conventional surgical procedures (2). Many digital applications and devices are aiding healthcare professionals in monitoring patients' real-time health status, even without visiting. After years of research, these digital devices are much more intelligent and sensitive and work based on the scientist's algorithm (3, 4). These devices are significantly increasing patients' recovery rates. Wearable devices manage the daily lifestyle routines of the users. The progress of digital technologies is changing the conceptualization of healthcare in recent times. Digital devices are nowadays mostly inbuilt functioning about the healthcare process and procedure.

Although technology and applications are sometimes not straightforward, many researchers developed user-friendly devices to enhance healthcare-related digital technologies. According to, digital healthcare significantly changed modern-day healthcare structure and made life easier for patients and healthcare providers. Despite the effectiveness of digital technologies in healthcare services, stakeholders reported several severe concerns about utilizing these technologies—for example, the security and safety of the patient's history. In digital health records, detailed information and history are available online, and they may not be secure from a privacy point of view.

Blockchain technologies are being introduced to overcome this challenge and considerably improve the security issues (5, 6); however, it is still in its infancy, and applications are minimal. Thus, a fundamental question that needs to be addressed here is what type of digital technologies are effective in the healthcare sector and how digital technologies have shaped the future landscape of digital healthcare? We understand that the penetration of digital technologies in the healthcare sector can't be effective unless interdisciplinary efforts have been made to provide relevant technology development. For this reason, we also aimed to map literature from a multidisciplinary perspective to highlight potential pitfalls and prospects.

This study is divided into five sections: the first section develops the background of the research and explains its goals; the second section talks about the research approach applied in this study; the third section highlights the key results, such as descriptive analysis, in-depth content and bibliometric analysis; the fourth section explains the results, specifically the four classes of digital technologies in healthcare; and the last section talks about conclusion, recommendations and limitations of the study.

## Literature on digital technologies in healthcare

Developments in digital technologies in healthcare provide an opportunity to provide uninterrupted healthcare services. The use of digital healthcare systems has benefited monitoring, diagnosis, prevention, and treatment (7, 8). Kapoor et al. (9) demonstrated many digital applications useful for digital health purposes during the pandemic. Rojas et al. (10) highlighted the use of internet-based programs in curing depression. Henkenjohann (11) evident that using patients' digital records improved healthcare services efficiency. Modern health records use blockchain technology to exchange electronic health records between patients and doctors (12).

Robotic surgery based on artificial intelligence helps doctors deliver personalized therapy to patients, eliminate repetitive activities, and prevent significant illnesses (13). However, Artificial intelligence (AI) applications create a tangle of legal issues for healthcare professionals and technology developers, especially if they cannot define AI-generated suggestions (13). Zimmermann et al. (14) provided meta-analytical evidence on the efficacy of eHealth interventions in supporting the emotional and physical wellbeing of people with type 1 and type 2 diabetes and comparing glycemic control and psychosocial support interventions.

While most academics have found evidence of digital technology's efficiency in healthcare systems, a minority have found conflicting outcomes (7). For example, Rojas et al. (10) findings indicated that the intervention should be improved by raising levels of personalization and implementing metrics to promote adherence. They reported mixed results in Chile and Colombia and highlighted the relevance of factors other than the content of the intervention, such as the intervention's location or context. There has been an increase in the usage of digital technologies in digital patient records. According to Henkenjohann (11), integrating an electronic health record offers potential benefits and risks an individual's privacy. Individual motives based on feelings of volition or external requirements influence digital technologies in healthcare adoption, even though internal incentives are more substantial. Blockchain technologies got attention from the practitioners to avoid the concerns raised by the researchers (15). However, blockchain technologies are still in the infancy stage, and many security and environmental concerns question using these technologies in healthcare.

The above discussion can be concluded in the disagreement of the researchers on the effectiveness of a one-fit solution for digital technologies in healthcare services (16). A thorough mapping of existing literature on these digital technologies concerning their efficacy and pitfalls must be done to highlight the potential improvements.

## Materials and methods

**Figure 1:**
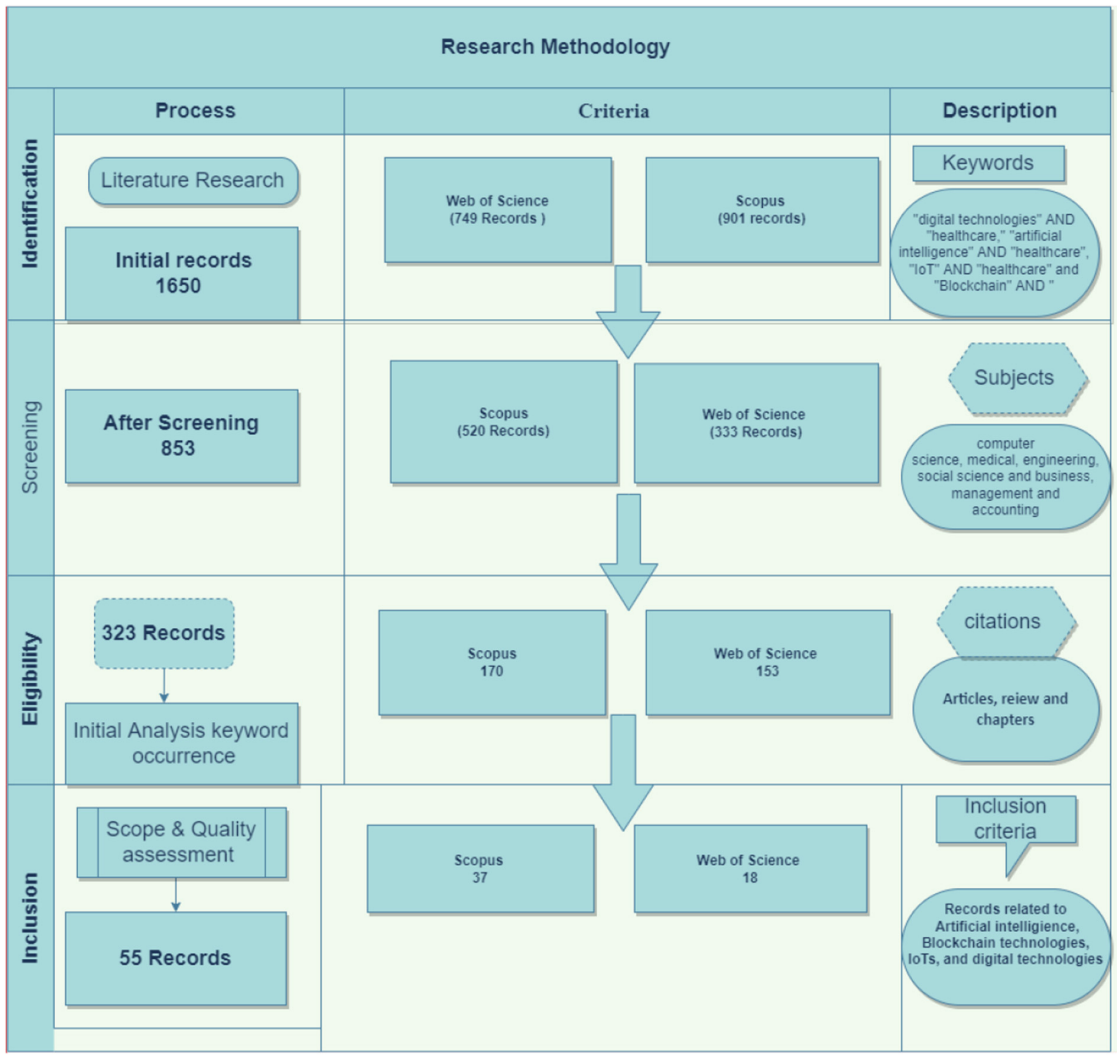
Review methodology.

The current research encompasses literature from two large, reputed databases, Scopus and Web of Science, among the researchers worldwide. We used “digital technologies” AND “healthcare,” “artificial intelligence” AND “healthcare,” “IoT” AND “healthcare,” and “Blockchain” AND “healthcare” keywords for the literature search. Initially, 1,650 records were obtained. The PRISMA framework was used to screen the records as suggested by Moher et al. (17) and shown in Figure 1. Critical inclusion and exclusion criteria used for this review were published articles in the English language and related to the digital technologies' scope in healthcare. The review papers, conference papers and review papers are excluded. Conclusive 323 studies are selected for stage 1 and used for keyword cloud and keyword occurrence. Later, a careful screening was performed for each identified classification to determine relevant records and only 55 articles were selected to be included to synthesize the review. Figure 1 shows the overall PRISMA statement selection and rejection process of the current study in detail.

## Results

### Descriptive analysis

**Figure 2:**
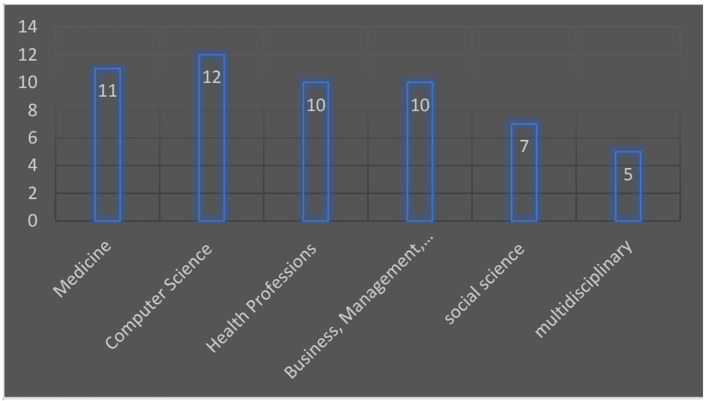
Showing the results of the subject.

Figure 2 shows the research question's multidisciplinary nature and highlights the different disciplines' contributions to emerging healthcare technologies. The most contributing field is computer science with 23.95% of studies included in the review, followed by the medical field with 22.01% of studies, engineering contributes 15.05% of studies and the combined contribution of social science and business, management and accounting is 8.74%, rest of the contribution is from different fields of studies like health profession, mathematics, decision science, biotechnologies, etc.

The records extracted from 1997 to 2021 and the Year-based publication and citation status are shown in Table 1. It is essential to assess the impact of digital technologies in healthcare research. Table 1 indicated the growing increase in published articles and citation count each year, with the highest frequency of publication and citation count in 2020. A total of 93 articles were records (28.79%) and 254 citations (16.57%).

**Table 1:** Publication and citation count.

**Year**	**Records**	**% of total records**	**Cited by**	**% of Total citations**
1997	2	0.62	40	2.61
2001	2	0.62	1	0.07
2004	3	0.93	3	0.20
2005	3	0.93	55	3.59
2006	4	1.24	23	1.50
2007	4	1.24	1	0.07
2009	5	1.55	27	1.76
2011	5	1.55	2	0.13
2012	6	1.86	38	2.48
2013	8	2.48	39	2.54
2014	8	2.48	33	2.15
2015	12	3.72	50	3.26
2016	22	6.81	204	13.31
2017	31	9.60	176	11.48
2018	45	13.93	449	29.29
2019	38	11.76	131	8.55
2020	93	28.79	254	16.57
2021	32	9.91	7	0.46
Grand total	323	100	1,533	100

**Figure 3:**
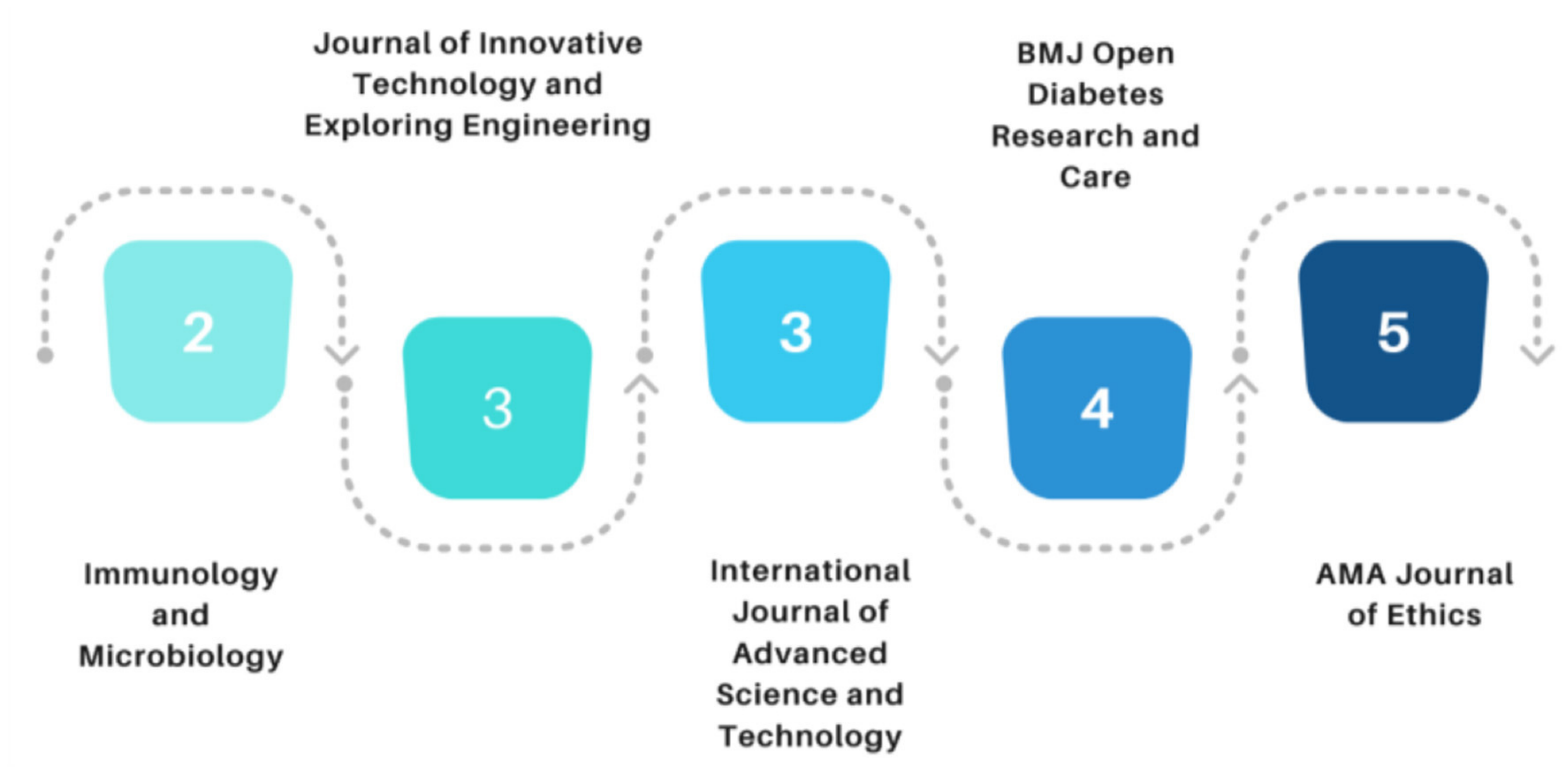
Journals with the most frequent publication in digital technology in healthcare.

Furthermore, the journal-based publication analysis is conducted for the current study and finds the AMA Journal of Ethics with the five publications. Second, most papers for this review were selected from the BMJ Open Diabetes Research and Care and Social Science and Medicine with 4. The study's name is gradually decreasing for the current study—International Journal of Advanced Science and Technology contributing 3 with International Journal of Innovative Technology and Exploring Engineering. Figure 3 shows the results of the research article selected from each journal.

### Literature classifications

Technological innovation is growing continuously, and researchers are looking deep into these technological changes step by step. Different technologies are used in healthcare development in the technological era—the current study evaluates the technology utilization for the healthcare sector. Further classification of technologies drives from the literature and researcher perspective toward technology adaptation in the healthcare sector. The digital technologies literature discusses mainly research for the development of healthcare. We used the keyword clouding technique to identify the most frequent keywords used in the studies. As mentioned above, there were 323 studies included in the keyword clouding technique at literature review stage 1; further, these studies were used to identify the literature classifications from these keywords, as shown in Table 2.

**Table 2:** Keyword occurrences and relevance score.

**Literature classification**	**Keywords**	**Occurrences**	**%**	**Relevance score**
Artificial intelligence (AI) & Machine learning	Disease	52	2.61	0.658
	System architecture	49	2.45	0.6254
	Efficiency	32	1.60	0.423
	Improvement	32	1.60	0.2623
	Doctor	26	1.30	0.3636
	Physician	26	1.30	0.5517
	Rehabilitation	25	1.25	5.5655
	Healthcare professional	24	1.20	0.7886
	Scale	19	0.95	0.4882
	Chronic disease	17	0.85	0.9735
	Patient care	16	0.80	0.9082
	Cloud	15	0.75	0.5318
	Machine learning	15	0.75	0.7005
	Healthcare sector	14	0.70	0.3983
	Practitioner	12	0.60	0.7772
	Emergence	11	0.55	0.4162
	**Total**	**385**	**0.1926**	**14.432**
Blockchain	Privacy	44	2.20	0.3005
	Blockchain	41	2.05	0.4962
	Effectiveness	37	1.85	0.4303
	Security	37	1.85	0.4581
	Algorithm	36	1.80	0.4369
	Performance	35	1.75	0.4548
	Artificial intelligence	34	1.70	0.261
	Experience	31	1.55	1.0292
	Big data	28	1.40	0.3102
	HER	18	0.90	0.6217
	Trust	18	0.90	1.0306
	EMR	13	0.65	1.926
	**Total**	**372**	**0.186**	**7.7555**
Digital technologies	Digital devices	117	5.86	0.2639
	Digital app	87	4.36	0.83
	Healthcare system	67	3.36	0.3188
	Internet	65	3.26	0.5561
	Innovation	60	3.01	0.7567
	Healthcare industry	28	1.40	1.293
	Digital health	26	1.30	0.3053
	Digital transformation	26	1.30	1.5063
	Analytic	25	1.25	0.3852
	Digital machine	22	1.10	0.4235
	Smartphone	22	1.10	0.8133
	Telemedicine	22	1.10	0.5556
	Medical device	21	1.05	0.5876
	Communication technology	19	0.95	0.3988
	Chatbot	16	0.80	1.6736
	E-health	16	0.80	0.9983
	Interview	16	0.80	4.6255
	Government	15	0.75	0.3507
	Digital platform	14	0.70	1.6069
	Digitalization	14	0.70	0.305
	Healthcare organization	13	0.65	0.764
	Digital health intervention	12	0.60	3.7538
	Digital revolution	11	0.55	0.5421
	RPD	11	0.55	2.8947
	**Total**	**745**	**0.373**	**26.5087**
Internet of Things (IoT)	Network	74	3.71	0.3104
	Implementation	60	3.01	0.5011
	IoT	58	2.91	0.8009
	System integration	48	2.40	0.4051
	Sensor	48	2.40	0.5065
	Internet of thing	45	2.25	0.7049
	Training	31	1.55	1.281
	Clinician	24	1.20	0.9669
	Information technology	16	0.80	0.4429
	Medical service	15	0.75	0.3193
	ICT	13	0.65	1.668
	TRAK	10	0.50	8.0116
	Interoperability	15	0.75	0.3749
	**Total**	**457**	**0.2288**	**16.2935**
	**Grand total**	**1,959**	**0.9804**	**64.9897**

**Figure 4:**
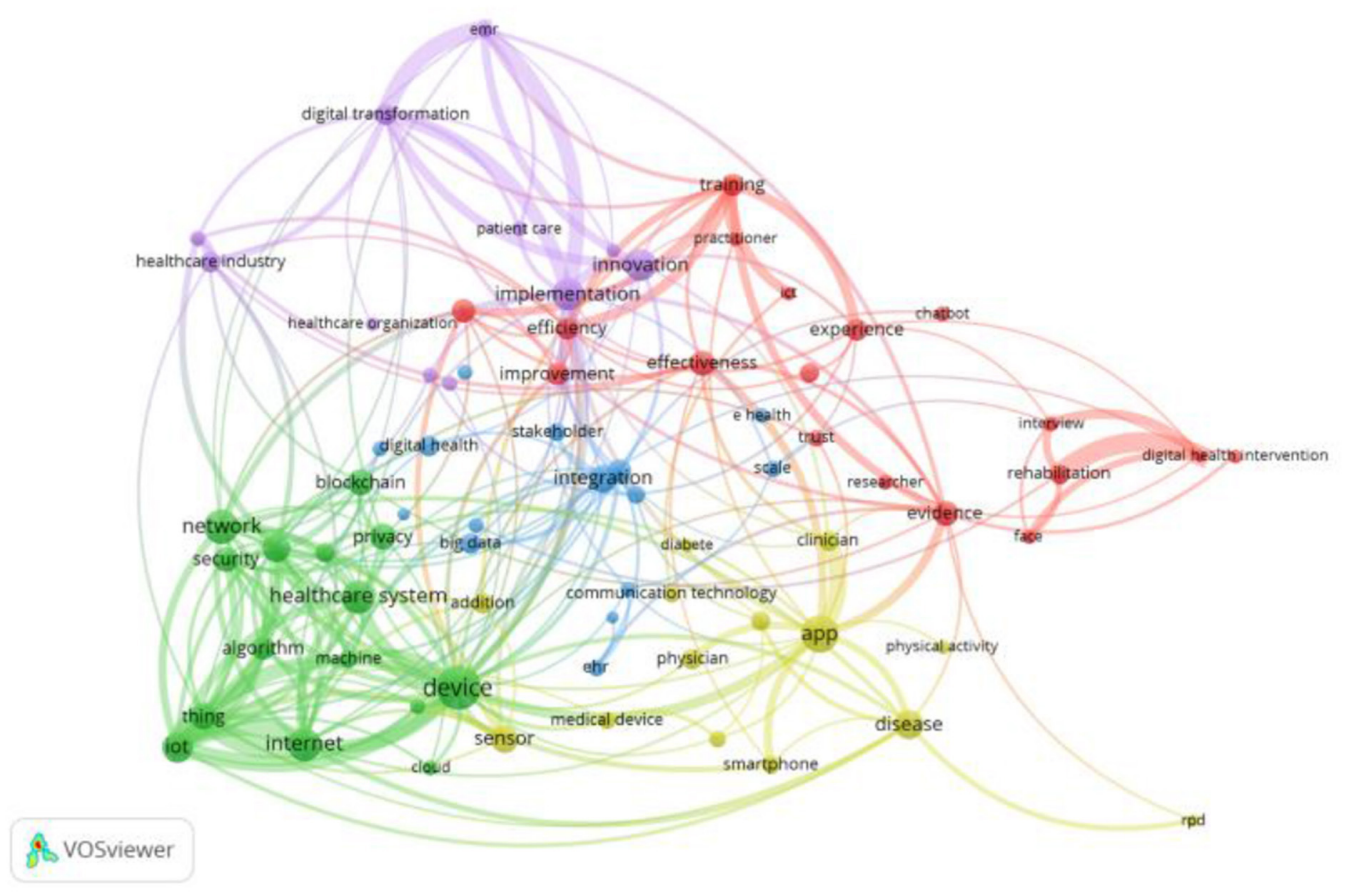
Co-occurrence of terms.

A selection of sixty-five most frequent keywords from 323 studies were conducted to identify the literature classifications. The keywords' occurrence and relevance scores were calculated using a text network using VOSViewer software and presented in Table 2. We also verified results obtained from the keyword clouding using the co-occurrence of the terms provided in Figure 4. We identified four major literature clusters on digital technologies in the healthcare sector based on co-occurrence and keyword clouding. The first cluster was named the application of digital technologies in the healthcare sector. The second is related to applying blockchain technology in healthcare; the third is Artificial Intelligence (AI) & Machine learning, and finally, using Internet-of-Things (IoT) in healthcare services. The following section provides more details about prospects and obstacles for each classification.

#### Application of digital technologies in healthcare

Digital technology's introduction in the healthcare sector positively indulges practitioners and patients. Devices, applications, and software are essential in healthcare, and Digital technologies have huge infrastructural and adaptation expenditures. However, the monitoring of the distance patients is valuable. Marent et al. (18) study findings are on HIV patients living in distant areas, and ambivalence technologies are used to send patients alerts. Studies conclude that ambivalence can counterweight passive and positive reports of technology and assist social researchers in bringing up their vital role inside the structure of digital health involvements.

Pirhonen et al. (19) use the model to enhance health-related awareness and care in old age people. Digital alarms and messages are creating more relevant services for old age people. They are easily monitored using digital devices. Simultaneously, the usage of digital devices in older people is insignificant due to the applications' complications. Results show that self-care is positively related to the patients. Due to the technology penetration, practitioners are more comfortable following up on the patients' historical background using digital devices. Digital health policy renders the patients' healthcare structure with the help of applications and online services. Enhancing self-care using digital technologies is vital in recent times, and pressure on traditional medical services narrows down. In the review, Joyce (20) suggests using textiles and medical devices in hospitals and homes. The baby band will replace the cardiopulmonary monitor in neonatal intensive treatment units to replace the belly band and fatal heart rate monitor during labor and birth in hospitals. Assessment of prospective operators' opinions of smart textiles confirms the modern forms of medicalization and reconnaissance medication. Smart textile medical devices, therefore, are keen on more significant developments in health care. Hospitals are constructed to be homelike and comfortable simultaneously as patients and instruments become fully open to data systems.

However, the technology driving skill is a barrier, and governments must apply policy for practitioners to learn better development in the healthcare sector. Monitoring distance patients through digital technologies is a more significant challenge for practitioners due to their skills and ability. Basholli et al. (21) investigate healthcare professionals' attitudes toward the application of distant patient monitoring via sensor networks in emerging areas using semi-structured interviews. The study's findings recommend that training and learning can develop the understanding of healthcare's digital platforms and help practitioners adopt the technologies.

Table 3 briefly details the digital technology literature authors, settings, procedures, and findings. It is also vital to create the importance of digital healthcare in citizens for adapting and learning for complete understanding. Petersen et al. (25) study findings showed government policies and initiatives toward the digital technologies adaptation. The study draws the model that involves citizens in significant determinations regarding digitalization, its potential consequences, and the primary independent shortage that this signifies. Another critical research also highlights the recent outbreak of the COVID-19 pandemic in the literature about the digital technologies' role in screening the infected people and monitoring the epidemic progress in hospitals to measure the actual numbers. The study uses the assisted living (AL) model for measuring threats. The study's findings summarize a few tests AL people encounter in their effort to follow COVID-19 state regulations built for lengthy-time care capabilities. According to Tortorella et al. (22), study findings conclude that adopting digital technologies is easy and efficient for developed countries and barriers to transforming technologies in low-income countries.

**Table 3:** Digital technologies.

**References**	**Process**	**Settings**	**Findings**
Tortorella et al. (22)	Skill full labor	Practitioners and patients	Results conclude that digital technologies adaptation is easy and efficient for the skilled labor force countries while having barriers in transforming technologies with low-income generating countries.
Ryhtä et al. (23)	Infrastructure	Devices	Digital technologies using skills are one of the critical learning in recent times.
Marent et al. (18)	HIV patients	Ambivalence technologies	HIV patients live in distant areas, and ambivalence technologies use to send alerts to the patients.
Pirhonen et al. (19)	Aged people	Digital alarm and messages	Results show that self-care is positively related to the patients
Petrakaki et al. (24)	Distance patients	Skills and ability	Monitoring distance patients through digital technologies is a more significant challenge for practitioners due to their skills and ability.
Basholli et al. (21)	healthcare professionals'	Distant patient monitoring	The findings of the study recommend that training and learning can develop the understanding of digital platforms in healthcare and help practitioners adopt the technologies
Joyce (20)	Bellyband	Birth in hospitals	Suggesting the use of textiles and medical devices in hospitals and homes.
Petersen et al. (25)	Government policies	Digital technologies adaptation	Findings showed that government policies and initiatives toward the digital technologies adaptation
Yang et al. (26)	COVID-19	Assisted living (AL) model	Summarize a few tests AL people encounter in their effort to follow COVID-19 state regulations built for lengthy-time care capabilities

#### Application of blockchain technologies in healthcare

As the digital technologies adaptation and replacement in many fields are growing daily, the number of risks and insecurity related to the data is higher. Data-related security is one of the particular issues in recent times for technology users. Blockchain is a decentralized structural design where data are stored in the shape of blocks for administering, as presented in Table 4. The data should be transmitted from one individual to another with protection and modernized with an intelligent agreement in the blockchain. The healthcare sector's insurance management uses the blockchain to identify the authorized individual permission when the individual is determining. The electronic health record is critical because important and personal private information is on the record. Arunkumar and Kousalya (29) conduct a study. Electronic health record (EHR) is a digital system of patient health information that usually encompasses patient communication data, vital signs, medical history, and current and past treatment subcontracts to the cloud. The study suggests using the cloud-based blockchain, encrypting the data using an authenticated encryption algorithm for healthcare high electronic record management results. The recent studies primarily concern the electronic health record recommending using the blockchain for security.

**Table 4:** Blockchain research in healthcare.

**References**	**Process**	**Settings**	**Findings**
Shobana and Suguna (27)	Security	Technology users	The data related security is one of the very exceptional issues in recent times for technology users
Ariyaluran Habeeb et al. (28)	Insurance management	Authorized individual permission	The electronic health record is very critical due to significant and individual private information is on the record
Arunkumar and Kousalya (29)	Electronic health record (EHR)	Patient health information	Mainly concerned about the electronic health record is recommending using the blockchain for security.
Murugan et al. (12)	electronic health record	Technology proposes	The system also exchanges the electronic health record between patients and doctor
Kumari et al. (30)	WBAN	Blockchain technology	The study recommends the transfer of medical records of the patients on the network like staff, management, emergency department, and insurance
Chen et al. (31)	Searchable encryption	HER	The system for HER is developing using complex logic expressions and records in the blockchain; the index for search can use for searching for the data.
Christo et al. (32)	Model Quantum Cryptography	IoT devices	In the digital world, security issues are related to the Internet of things very much, and IoT devices are more at risk due to the nature of the work
Rathee et al. (33)	Hypermedia data	Security framework	It expects that the IoT is not secure for use, and many cyber-attack risks are associated with the devices due to the limited knowledge and skills of the users and system limitations
Qashlan et al. (34)	Transportation	Peer-to-peer networks	The findings of the study demonstrate three valuable blockchain tools access control and evaluation of the performance of the model
Kumar and Mallick (35)	Data secure	IoT	The study explains that In IoT, the switch of data and data verification is simply accomplished across the central server to the protection and secrecy fears.

Murugan et al. (12) propose a health information exchange solution using blockchain technology. The system also exchanges the electronic health record between patients and doctors; the system also operates in the healthcare aspect to safely improve insurance claims and data used by the research organizations. Another study in the review also contributes to maintaining the Electronic health record using the blockchain technology in WBAN. The study recommends transferring patients' medical records on the network like staff, management, emergency department, and insurance. Traditionally the security models use the centralized network in IoT. The study in the review proposes the decentralized, secure, and peer-to-peer networks model of blockchain technology to secure different fields like transportation, logistics, and healthcare. The study's findings demonstrate three valuable blockchain tools access control and evaluation of the model's performance. Kumar and Mallick (35) contribute a study to make the data secure and information flow. The study explains that In IoT, the switch of data and data verification is simply accomplished across the central server to the protection and secrecy fears.

Although authors have many different blockchain technology models for securely transferring and sharing patient records, many have raised concerns over data transfer security. The security issues in EHR are hazardous due to the nature of the information. Chen et al. (31) propose a searchable encryption blockchain system for EHR. The EHR system is developed using complex logic expressions and records in the blockchain; the search index can search for the data.

Cyber-attack risks are concentrating the intentions of blockchain technology on more adaptation in the electronic health record. The technology uses authentication, Encryption, and Data Retrieval in the short blockchain's electronic health record. For this purpose, Christo et al. (32) use a model Quantum Cryptography for Encryption—AES and Data Retrieval—SHA algorithms to avoid the numerous raids. In the digital world, security issues are related to the Internet of Things, and IoT devices are more at risk due to the work's nature. Rather et al. (33) provide a security framework of healthcare hypermedia data via the blockchain to counter this risk associated with the IoT devices. They are creating the middle of each data so that any changeover, variation in data, or medication contravening might show in whole blockchain system users. Usually, it expects that the IoT is not secure for use. Many cyber-attack risks are associated with the devices due to their limited knowledge, skills, and system limitations. Even though blockchain technology is a comprehensive tool for the security of the digital world and electronic records, significant challenges exist to blockchain adoption in healthcare. Technical challenges like processing speed and massive data duplication are still obstacles to blockchain technologies in healthcare.

#### Application of artificial intelligence (AI) and machine learning

The data complexity and rise in the healthcare sector showing that AI is working in the healthcare field, as shown in Table 5. Many different types of AI services have been rendered in the healthcare sector recently. According to Agarwal et al. (36), artificial intelligence and robotic surgery allow practitioners to facilitate patients in personalized healthcare, decrease repetitive tasks, and move forward to prevent serious illness. The recent development in machine learning and artificial intelligence provides personalized care without the patient's differences. Chen et al. (43) study machine learning and artificial intelligence findings, evaluating and distinguishing different artificial intelligence effects in healthcare and using a machine learning algorithm on unstructured clinical and psychiatric explanations to calculate an intensive care unit (ICU) death. Artificial intelligence (AI) application uniquely presents complicated issues concerning healthcare professionals and technology manufacturers' obligations if they cannot describe suggestions created by AI technology. For the quality of care and low down, healthcare AI must be using the troublesome effect. Physicians need to learn to work correctly with the system for effective working, as the electronic health records do. Physicians will need to realize AI techniques and procedures appropriate to confide in an algorithm's calculations.

**Table 5:** Artificial Intelligence (AI) & machine learning in healthcare.

**References**	**Process**	**Settings**	**Findings**
Davenport and Kalakota (4)	AI services	Healthcare field	The data complexity and rise in the healthcare sector showing that AI is working in the healthcare field.
Agarwal et al. (36)	Robotic surgery	Serious illness	Artificial intelligence and robotic surgery make it possible for practitioners to facilitate patients
Sullivan and Schweikart (37)	Machine-learning algorithm	Intensive care unit (ICU)	Findings of machine learning and artificial intelligence evaluating and distinguishing different effects of artificial intelligence in health care.
Neubeck et al. (37)	Legitimate issues	Application of artificial intelligence	The application of artificial intelligence (AI) presents complicated legitimate issues concerning healthcare professionals and technology manufacturers' obligations, uniquely
Crigger and Khoury (38)	Troublesome effect	Electronic health records	Physicians will need to realize AI techniques and procedures appropriate to be competent to confide in an algorithm's calculations
Garbuio and Lin (39)	AI start-ups	Entrepreneurs in the healthcare	AI largely depends on the skill of technology physicians use, and many governments are looking to advance the learning.
Tang et al. (40)	Skill of technology	Job efficiently	Physicians must learn to do a job efficiently with artificial intelligence systems
Laï et al. (41)	Healthcare companies	Algorithms medicine precision	Technology usage in healthcare is a novel idea in recent times, specifically the algorithms to predict the medicines for the patients.
Wartman and Combs (42)	Doctors' skill	(AI) applications	That big collective data produces analytical and treatment endorsements and allocates self-assurance assessments to those endorsements.

The last decade are empowering technology and new start-ups that are changing the overall marketplace. Big ventures are investing in technology-based innovations to provide solutions for customers and manufacturers. Garbuio and Lin, (39) article investigates a real-time critical analysis of the AI start-ups model. It brings a solution for the entrepreneurs in the healthcare sector in the world. AI largely depends on physicians' technology skills, and many governments are looking to advance learning. To improve the healthcare promise by using AI to promote quality of care and minimize the adverse effects. Physicians must learn to do a job efficiently with artificial intelligence systems. However, according to reports, AI is using 86% of healthcare companies in some form. The top listed applications of AI in healthcare are predictive algorithms and precision. That helps predict patients' risks, correctly diagnose, prescribe drugs, and still concentrate on maintaining or allocating restricted wellbeing assets. In recent times, technology usage in healthcare is a novel idea, specifically algorithms to predict the patients' medicines.

Many researchers firmly believe that the future of healthcare is related to AI and machine learning due to their positive contribution to healthcare. However, researchers are also concerned about the ethical considerations related to the usage of AI in Healthcare. Existing health check experience beats the human mind's coordinating capability, yet medical education continues cantered on knowledge procurement and treatment. According to Wartman and Combs (42), Confusing this excess data disaster between apprentices is the circumstance that doctors' skill sets now must include cooperating with and dealing with artificial intelligence (AI) applications. That big collective data produces analytical and treatment endorsements and allocates self-assurance assessments to those endorsements. Legitimate specialists and industrial designers of AI implement that assistance in identification must also start to tackle responsibility issues when inaccurate diagnoses are affected by a human being using AI tools directly. Questions also remain regarding the changing role of the understanding-physician association and fiduciary agreement in an algorithm-enabled healthcare environment—Table 5 shows complete details of authors, process, settings, and findings.

#### Application of internet of things (IoT) in healthcare

Growing wireless communication, digital electronic devices, and microelectronic mechanical systems technologies represent the Internet of Things (IoT) evolution. In comparison, IoT components are smartphones, tablets, laptops, wearable devices, electric household appliances, and Wi-Fi devices. Due to effectiveness, the healthcare sector is also moving very quickly in recent years toward IoT devices. The healthcare of society and technology relationship is building due to the Internet of things with numerous networking capabilities. According to Abdelgawad et al. (44), IoT is used to interconnect the best possible resources, look at inefficient resources, and offer efficient and reliable intelligent medical care services to aged people. Improve the elderly lifestyle, and these devices are an advantage for active and quality living. However, health-related data processing is vital in healthcare and carries critical issues like security and authentication. Jeong et al. (45) proposed a protocol that offers construction in multi-dimensional color for the patients and users associated with managing their condition in different groups.

Besides that, Sangeetha et al. (46) study conducted the changes and challenged India's healthcare system with life-threatening diseases and recent pandemic outbreaks like COVID-19. The study's findings conclude that the government needs to use the accessibility and affordability of health care, human resource, infrastructure development, e-health, and IoT (Internet of things) technology in the healthcare sector. The IoT is growing increasingly in the healthcare system and is also challenging the security concerns of patients in healthcare. Managing massive quantity data such as reports and pictures of every individual indicates improving individual attempts and security threats. Rathee et al. (33) manuscript to overcome the security threats is more valuable. Table 6 shows the authors, year, methodology, process, and setting details related to healthcare IoT devices. Qashlan et al. (34) findings are also related to security and privacy are recommending blockchain technology.

**Table 6:** Internet of things (IoT) in healthcare.

**Author**	**Process**	**Settings**	**Findings**
Parimi and Chakraborty (47)	Wireless communication	Patient data	The main idea is to record the historical background, present, and future are to use the control, communicate, store, and recover the patient data to provide focus health-related services
Javed et al. (48)	Wireless communication	Internet of Things (IoT)	While the components of IoT are smartphones, tablets, laptops, wearable devices, electric household appliances and Wi-Fi devices
Abdelgawad et al. (44)	Medical care services	Elderly lifestyle	The study author, based on data collection and analysis, offers a prototype for architecture for performance advantages
Jeong et al. (45)	IoT devices	Security concerns	Most researchers highlight the security concerns related to medical devices and IoT in the current review.
Arfaoui et al. (49)	Wireless Body Area Network	Unknown verification method	From a security viewpoint, the recommended method completes privacy, reliability, secrecy, perspective-aware privacy, key escrow challenge, people verifiability, and ciphertext accuracy
Sangeetha et al. (46)	Healthcare system	Life-threatening disease	The study also concluded that digital penetration is more effective in healthcare in primarily populated states.
Rathee et al. (33)	Security threats	Privacy and security	In directive to avoid these problems, Blockchain technology has been combated as the safest method that offers the privacy and security of self-control structure in actual time circumstances
Qashlan et al. (34)	Security and privacy	Blockchain technology	Findings are also related to security and privacy are recommending the blockchain technology

The IoT devices growth is increasing in medical health services very rapidly. Security and privacy concerns are some of the primary issues associated with IoT and digital devices. Arfaoui et al. (49) pinpoint the Wireless Body Area Network (WBAN) related study to handle these issues. The context-conscious gain access to self-control and unknown verification method cantered on a safe and effective Hybrid Certificateless Signcryption (H-CLSC) program. The recommended process https://www.sciencedirect.com/topics/computer-science/achieve-confidentiality, reliability, secrecy, perspective-aware privacy, key escrow challenge, people verifiability, and accuracy from a security viewpoint.

## Conclusion and discussion

**Figure 5:**
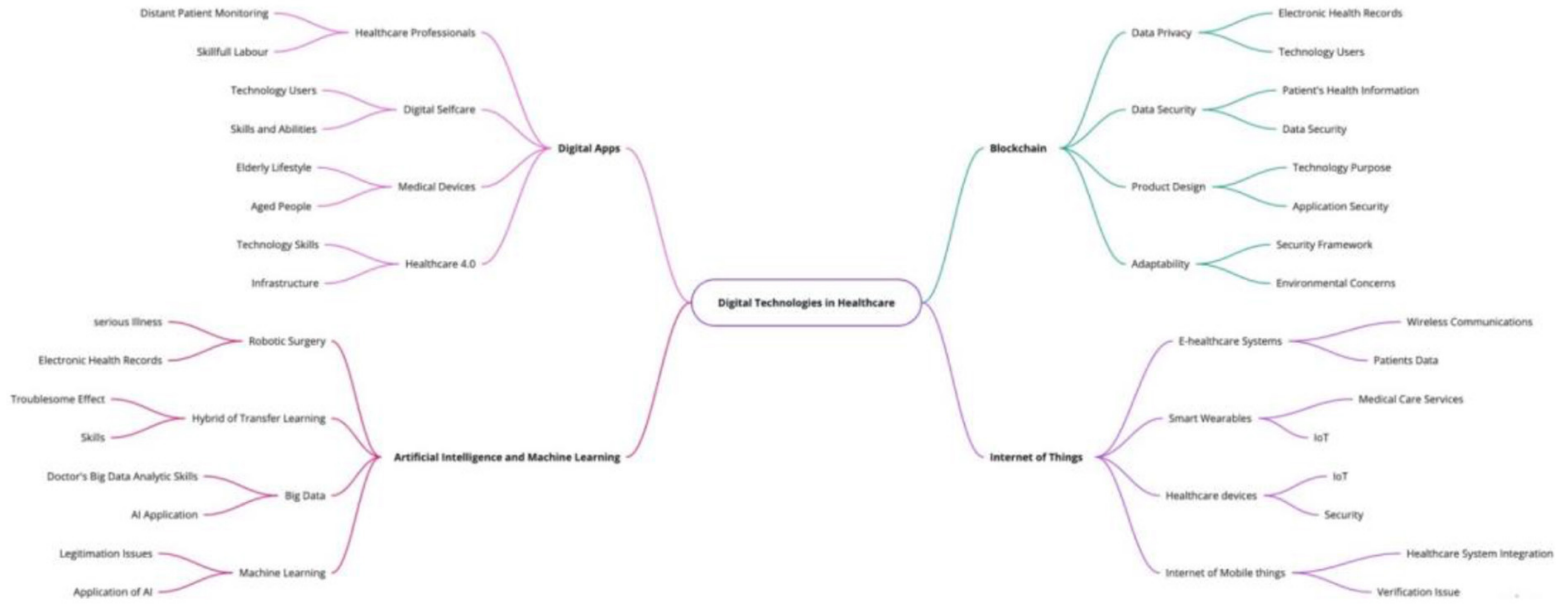
Mapping of literature on technologies in healthcare.

Technology development provides a toolbox that enhances patient care models and boosts patient management services and safety, improving approachability, and accuracy in all health areas. Findings of the review on technological developments in healthcare research have exposed four major classifications of the literature, as shown in Figure 5. Traditional medical care is disruptive through telemedicine, digital mobile health, applications, artificial intelligence, and other Internet of things. The conventional mediums are replacing these mediums primarily during this century. Technology adoption in healthcare is remarkably developing healthcare. Digital technologies are making more natural processes in healthcare. The literature in the current review discusses the skills and capabilities to use digital technologies more critically. Many new technologies can be learned quickly, and some are difficult due to the nature of jobs in the healthcare sector. For improving the skills and abilities, pieces of training are essential for development. Besides, online medical services and applications feature the demand and effectiveness at a higher ratio due to digitalization in healthcare.

As summarized in Figure 5, digital applications make dealing with minor health issues more accessible, and digital technologies significantly contribute to older adults' health issues. Elderly patients are usually in very critical health issues, and traditionally hard to manage their health records. However, electronic health record-keeping the history of patients. Electronic health record systems are significantly contributing to modern-day healthcare. At the same time, some issues related to digital technologies used in healthcare. Many studies concern the use of digital technologies, and IoT devices involve data security risks. However, several contributions are associated with digital technologies but hard to avoid the privacy records in an electronic health record.

Blockchain technologies are a better and more secure option to manage patient data safety in a digital technology-based healthcare system. Researchers are proposing many robust models and manuscripts to keep the data safe. The real challenge in eHealth is keeping patients' records and history safe. The number of healthcare systems using companies is adopting blockchain technologies instead of main server networks. That creates more reliability and authentication for secure data management. In the current study, blockchain-related literature commonly contributes to the safety and security of vital patient data in blockchain technologies. The number of Internet of things (IoT) devices is growing as the technology penetration in the healthcare system is growing. Smartphones, tablets, laptops, wearable devices, electric household appliances, and Wi-Fi devices are examples of IoT. Fast-going lifestyle is making it more compulsory for the users to adopt these smart devices to manage their job and business affairs, and healthcare dependencies are moving on these devices. IoT devices are commonly prevalent in every age. Researchers believe that the number of devices growing in healthcare will make it easier for healthcare systems to deal online, and the load will decrease. The instruments and research are gradually improving the quality of health services; these devices' significance is much higher. Finally, artificial intelligence and machine learning in healthcare is very effective and dominant due to their significant features. AI is increasing in the healthcare management systems, and physicians are replacing AI machines to handle patients' issues. Robotic surgeries are very effective in the modern-day medical healthcare system, and the future of healthcare is related to machines and robots. Highly effective and equipped robots will replace the physicians in operation theaters.

## Data availability statement

The datasets presented in this study can be found in online repositories. The names of the repository/repositories and accession number(s) can be found in the article/supplementary material.

## Author contributions

NA secured article processing charges to facilitate the publication of the research article. MQ and NK were responsible for conceptualizing the idea, manuscript preparation, and data analysis. SQ reviewed and amended the prepared manuscript. SH contributed to the revised manuscript. NK was also responsible for data curation and exporting from relevant databases. All authors contributed to the article and approved the submitted version.

## Funding

This work was supported by the Guangdong Social Science Project (Grant No. GD21CSH07).

## Conflict of interest

The authors declare that the research was conducted in the absence of any commercial or financial relationships that could be construed as a potential conflict of interest.

## Publisher's note

All claims expressed in this article are solely those of the authors and do not necessarily represent those of their affiliated organizations, or those of the publisher, the editors and the reviewers. Any product that may be evaluated in this article, or claim that may be made by its manufacturer, is not guaranteed or endorsed by the publisher.
